# Maternal intraguild predation risk affects offspring anti-predator behavior and learning in mites

**DOI:** 10.1038/srep15046

**Published:** 2015-10-09

**Authors:** Michael Seiter, Peter Schausberger

**Affiliations:** 1 Group of Arthropod Ecology and Behavior, Department of Crop Sciences, University of Natural Resources and Life Sciences, Peter Jordanstrasse 82, 1190 Vienna, Austria

## Abstract

Predation risk is a strong selective force shaping prey morphology, life history and behavior. Anti-predator behaviors may be innate, learned or both but little is known about the transgenerational behavioral effects of maternally experienced predation risk. We examined intraguild predation (IGP) risk-induced maternal effects on offspring anti-predator behavior, including learning, in the predatory mite *Phytoseiulus persimilis*. We exposed predatory mite mothers during egg production to presence or absence of the IG predator *Amblyseius andersoni* and assessed whether maternal stress affects the anti-predator behavior, including larval learning ability, of their offspring as protonymphs. Protonymphs emerging from stressed or unstressed mothers, and having experienced IGP risk as larvae or not, were subjected to choice situations with and without IG predator traces. Predator-experienced protonymphs from stressed mothers were the least active and acted the boldest in site choice towards predator cues. We argue that the attenuated response of the protonymphs to predator traces alone represents optimized risk management because no immediate risk existed. Such behavioral adjustment could reduce the inherent fitness costs of anti-predator behaviors. Overall, our study suggests that *P. persimilis* mothers experiencing IGP risk may prime their offspring to behave more optimally in IGP environments.

Predation risk is a strong selective force shaping prey morphology, life history and/or behavior^1^. Anti-predator behaviors enhance prey survival but are inevitably traded-off against other activities such as feeding, mating or egg production. Due to the inherent costs of any anti-predator activity, prey should be able to distinguish between different levels of predation risk and adjust their behavior accordingly^2^, as predicted by the threat-sensitive predator avoidance hypothesis^3^,^4^.

During life, most prey individuals encounter multiple predator species, which compete for prey. Predation among species competing for the same resources is called intraguild predation, IGP^5^. IGP differs from classical predation in that IG predators may not only obtain nutrients but at the same time eliminate potential predators and resource competitors. Mutual IGP is very common among plant-inhabiting predatory mites of the family Phytoseiidae, the focal animals of our study, but unbalanced and life stage-specific^6^. Adult females are relatively invulnerable to IGP^6^. Larvae are the most vulnerable IG prey because of their limited mobility and limited defensive abilities. Larvae may themselves reduce the risk of IGP^7^, but predominantly their mothers decrease offspring IGP risk via selective egg placement and/or killing potential future offspring predators^8^. Mothers could also prepare the embryos such to better cope with predation risk after birth. For example, stickleback mothers transfer information about predation risk to their eggs, influencing the eggs’ nutritional and/or hormonal status and thereby offspring development and behavior^9^. Such effects are generally dubbed maternal effects and defined as phenotypic effects of mothers altering the offspring phenotype but not genotype^10^,^11^. Typical environmental factors inducing maternal effects are food availability, social context and predation risk. Maternal predation risk can influence offspring anti-predator behaviors^12^, morphologies^13^ or development^11^. For example, bird mothers have elevated stress hormone levels when predators are present^14^, which may negatively affect offspring body size as a whole yet positively affect wing growth rates^15^. Accordingly, we firstly hypothesized that offspring of predatory mite mothers encountering IG predators are better able to respond towards predation risk than are offspring of unexperienced mothers.

Learning is a ubiquitous phenomenon in animals, allowing individuals to adjust their behaviors to variable environments^16^. Learning may affect every important life activity (such as foraging, mating, anti-predator behavior) but little is known about maternal effects, and here particularly those induced by predation risk, on offspring learning ability. A pertinent study with sticklebacks showed that maternal stress, caused by predator presence, may adversely affect offspring learning^17^. Offspring of predator-stressed mothers were less able to improve foraging by experience than were offspring of unstressed mothers. However, alternative possibilities in such scenarios are that maternal stress renders negligible learning by offspring, because they are already prenatally prepared, or, enhances learning by offspring, because they are already prenatally sensitized. Accordingly, we secondly hypothesized that the IGP risk experienced by predatory mite mothers affects, and may interact with, the learning ability of their offspring in IGP environments.

We pursued our hypotheses in the predatory mite species *Phytoseiulus persimilis,* which is a highly specialized predator of spider mites such as *Tetranychus* spp., as IG prey, and *Amblyseius andersoni,* which is a generalist predator of various mites and small insects^6^,^18^, as IG predator. *A. andersoni* is a strong and aggressive IG predator of co-occurring predatory mites such as *P. persimilis*^8^. In contrast*, P. persimilis* is a weak IG predator but often attacked as IG prey. In phytoseiid mites, gravid females are themselves not at IGP risk, but their offspring, especially the larvae, are highly endangered. Thus, presence of IG predators causes stress in ovipositing *P. persimilis* females on account of the future IGP risk of their offspring^7^,^8^. Juvenile development of phytoseiid mites proceeds from the egg through larva, protonymyph, deutonymph to adult. The six-legged larvae have low mobility and are in most species, including *P. persimilis*, non-feeding^6^. Protonymphs have eight legs and represent the first feeding stage of *P. persimilis*. Protonymphal IGP risk decreases with increasing age and size^6^,^7^. Under IGP risk, mothers may either innately or after experience select suitable oviposition sites, to provide for a safe place for hatching and development^8^,^19^. Additionally or alternatively, they could prenatally prepare their offspring, for example via nutritional and/or hormonal and/or epigenetic effects, to better cope with IGP risk after birth. Maternal effects influencing offspring anti-predator behavior and learning have rarely been assessed (see^12^,^14^,^17^ for birds and fish) and have never been looked at in arthropods such as predatory mites.

We investigated whether *P. persimilis* mothers internally modify the nutritional and/or hormonal and/or epigenetic status of their eggs under IGP risk posed by *A. andersoni* and thus induce phenotypic behavioral changes in offspring, including their ability to learn about predators. To this end, we first generated IGP-stressed and unstressed mothers, let their offspring in the larval stage experience IG predators or not, and then assessed their response as protonymphs towards IG predator traces in binary choice situations composed of sites with and without such traces.

## Methods

### Rearing of mites

*Phytoseiulus persimilis* and *Amblyseius andersoni* were reared in the laboratory on separate acrylic arenas. The laboratory populations were founded with specimens collected in Sicily^8^. Each rearing arena consisted of an acrylic plate (14 × 14 × 0.2 cm) placed on top of a water-saturated foam cube in a plastic box (20 × 20 × 6 cm) half-filled with tap water. Wet tissue paper was wrapped around the edges to establish a border between the acrylic plate and the surrounding water, and to prevent the predatory mites from escaping. Cotton wool fibers under coverslips served as shelters and oviposition sites for the predatory mites, *A. andersoni*; prey spider mites *T. urticae* were brushed from infested bean leaves onto the arena at two to three day intervals. *T. urticae* was reared on whole bean plants at room temperature. For *P. persimilis*, spider mite-infested bean leaves were piled up on the arena and new leaves added at two to three day intervals. The predatory mite rearing units were kept in environmental chambers at 25 ± 1 °C, 60 ± 5% RH, and 16:8 h L:D.

### Pre-experimental procedures

To test for maternal effects, ovipositing *P. persimilis* females were either exposed to IG predators representing a future IGP risk for their offspring, and thus causing maternal stress, (subsequently called “stressed”) or not exposed to IG predators (“unstressed”). IGP-stressed and unstressed females produced eggs giving rise to the experimental individuals, which themselves either experienced IG predators (subsequently called “predator-experienced”) or not (“predator-naïve”).

To generate stressed and unstressed *P. persimilis* females, groups of 10 adult females, randomly taken from the rearing unit, were placed on detached bean leaf arenas for 72 h and provided with a predefined number of *T. urticae* as prey. Each bean leaf arena (5 × 5 cm) consisted of a trifoliate bean leaf placed upside down on a water-saturated foam cube in a plastic box half-filled with tap water. Wet tissue paper was wrapped around the edges of the leaves to prevent the predatory mites and their prey from escaping. The number of *T. urticae* provided to the predators was determined in pilot experiments and represented enough prey for optimal oviposition of each *P. persimilis*^20^ and unlimited feeding by *A. andersoni* (if present), and at the same time, warrant frequent encounters between *P. persimilis* and *A. andersoni* (if present)^8^. Stressed *P. persimilis* females were generated on arenas with *A. andersoni*: two gravid *A. andersoni* females were added onto leaf arenas to establish an IGP environment that would be risky for *P. persimilis* offspring and therefore stressful to their mothers. Unstressed *P. persimilis* females were held on arenas without *A. andersoni*. Presence of co-occurring conspecific individuals does not represent a stressor for ovipositing *P. persmilis*, as compared to presence of the IG predator *A. andersoni*^8^,^21^,^22^. After 72 h, IGP-stressed and -unstressed *P. persimilis* females were placed singly in closed acrylic cages^23^ for 12 h to deposit eggs giving rise to experimental individuals. Most females laid only one egg during the 12 h period. The empty acrylic cages represented inert environments that contained no cues whatsoever of the IG predators and thus warranted that the eggs were only influenced internally by maternal provisioning.

To generate predator-experienced and –naïve offspring, eggs from unstressed and stressed *P. persimilis* females were taken out from the acrylic cages and placed in groups of ten on separate spider mite-infested bean leaf arenas, either with or without two gravid *A. andersoni* females. The number of *T. urticae* provided represented enough prey for optimal development of each juvenile *P. persimilis*^20^ and unlimited feeding by the IG predator *A. andersoni* (if present) but at the same time warranted encounters between *P. persimilis* and *A. andersoni* (if present)^8^. *P. persimilis* were left on these arenas until reaching the late protonymphal stage, which occurred after ~3 to 4 days. *Amblyseius andersoni* killed ~10 to 15% developing *P. persimilis* but the set-up provided for random IGP^8^. Maternal stress (yes/no) and offspring predator experience (yes/no) resulted in four groups of experimental individuals: (1) naïve offspring from unstressed mothers, (2) naïve offspring from stressed mothers, (3) experienced offspring from unstressed mothers and (4) experienced offspring from stressed mothers.

### Experimental procedure

In the choice experiment, we used acrylic cages^24^. Each choice cage consisted of two large cavities (Ø 1.5 cm) and a small cavity (Ø 0.5 cm), connected to each other with a T-shaped corridor, closed at the bottom by a mesh and on the upper side by a microscope slide^23^. Each experimental protonymph was offered a choice between the two large cavities each furnished with 20 *T. urticae* eggs, and one with and the other without IG predator cues. Choice cages were prepared one day before the experiment took place. Each of the two large cavities was loaded with 20 eggs of *T. urticae* using a moistened brush. The corridor was blocked by an inert plastic piece and a single adult *A. andersoni* female was introduced to one cavity, and allowed to feed and leave metabolic waste products and, possibly, chemical footprints for 16 h. To make the cages ready for experimental use, the *A. andersoni* female was removed, the number of *T. urticae* eggs was replenished to 20, and the corridor was opened by removing the blocking plastic piece. To start the choice experiment, single late (~1 to 1.5 days after molting) protonymphs from the four treatment groups were released in the small bottom cavity of the “T”-shaped maze and thus given the choice between a site with only *T. urticae* eggs or a site with *T. urticae* eggs and cues of the IG predator *A. andersoni*. The position of the protonymph (inside the cavity with or without predator cues or somewhere else, considered the neutral zone) and their activity (moving/stationary) were checked ten times, immediately after release and then every 20 min for three hours in total. Additionally, the number of individuals observed feeding was noted. Each choice unit and each experimental protonymph were used only once. Each treatment was replicated 20 to 22 times.

Using IBM SPSS 21 (IBM Corp., USA), the influence of maternal stress and larval predator experience on site choice and activity of the protonymphs (moving/stationary) was analyzed by generalized linear models [GLM^25^; binomial distribution (counts of events in sequence of observations) with negative log-log link for site choice and logit link for activity]. Model selection was based on Akaikes Information Criterion (AIC). Before analyses, the repeated observations were aggregated into one value for each individual.

## Results

**Figure 1:**
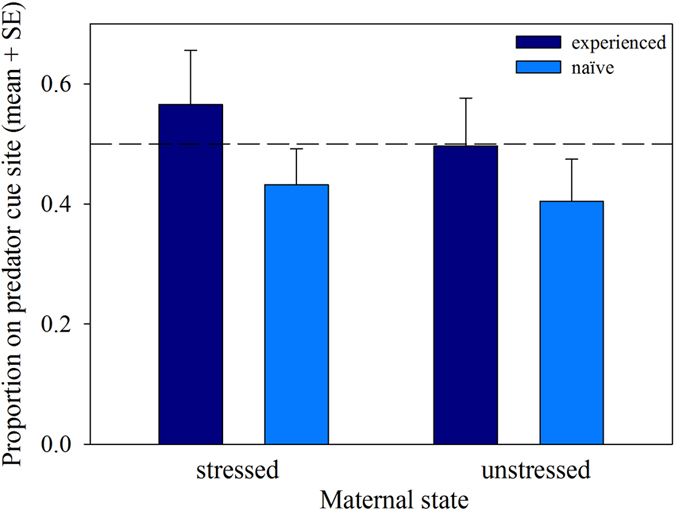
Site preference of predator-naïve and -experienced protonymphs originating from unstressed and IGP-stressed mothers. Protonymphs were given a choice between two sites, one with and one without cues of the IG predator *A. andersoni*. The broken horizontal line represents random residence. Both main factors, maternal state (stressed/unstressed) and IG predator experience, and their interaction had significant effects on site preference (GLM: P < 0.05).

**Figure 2:**
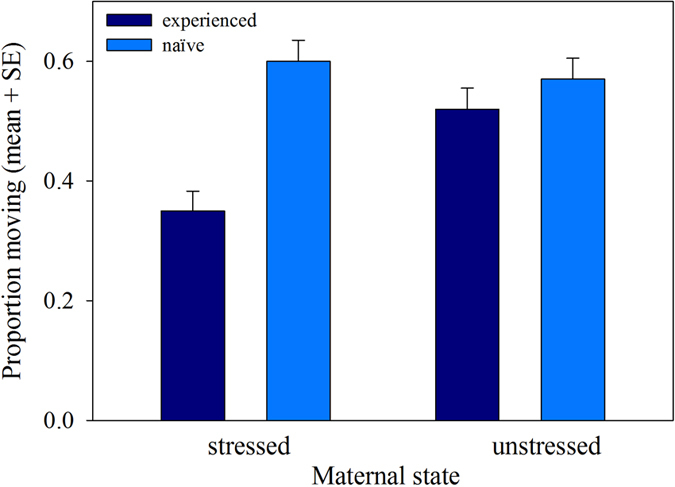
Activity (moving/stationary) of predator-naïve and -experienced protonymphs originating from unstressed or IGP-stressed mothers. Protonymphs were given a choice between two sites, one with and one without cues of the IG predator *A. andersoni*. Both main factors, maternal state (stressed/unstressed) and IG predator experience, and their interaction had significant effects on activity (GLM: P < 0.05).

Both maternal stress (GLM: Wald *χ^2^*_1_ = 4.764, P = 0.029) and IG predator-experience (Wald *χ^2^*_1_ = 10.583, P < 0.001) as main factors and their interaction (Wald *χ^2^*_1_ = 4.840, P = 0.028) influenced the site choice of *P. persimilis* protonymphs (Fig. 1). Protonymphs from stressed mothers and predator-experienced protonymphs resided more frequently in the predator cue site than protonymphs from unstressed mothers and predator-naïve protonymphs, respectively. The difference between protonymphs from stressed and unstressed mothers was more pronounced in predator-experienced than -naïve protonymphs (Fig. 1). Both maternal stress (GLM: Wald *χ^2^*_1_ = 3.782, P = 0.05) and predator experience (Wald *χ^2^*_1_ = 19.453, P < 0.001) as main factors had an influence on the activity (moving/stationary) of the protonymphs (Fig. 2). The lower activity of experienced than naïve protonymphs was more pronounced in stressed than unstressed mother, as indicated by the significant interaction (Wald *χ^2^*_1_ = 8.209, P = 0.004). Feeding incidences were generally rare, 4 to 6 individuals out of 20 to 22 per group were observed feeding, and similar among treatments.

## Discussion

Our study suggests that *P. persimilis* mothers experiencing IGP risk during internal egg formation prenatally influence the behavior of their offspring, including learning, in IGP environments. Both site choice and activity of *P. persimilis* protonymphs were affected by maternal stress and individual IGP experience. For both traits, site choice and activity, maternal stress during egg production interacted with individual learning by offspring. Predator-experienced protonymphs from stressed mothers were the least active and acted the boldest in site choice towards predator cues. It thus seems that protonymphs from stressed mothers had improved learning abilities because they were better able to discriminate the immediate risk previously associated with physical predator presence and the latent risk posed by predator traces alone.

During the learning phase, larvae and early protonymphs were exposed to high IGP risk (physical presence of predator females, their eggs and traces) whereas, in the choice situation, late protonymphs were exposed to sites with and without predator traces, indicating latent risk. Since the predator-experienced protonymphs acted bolder towards predator traces than the naïve ones did, our experiment suggests that learning allowed the protonymphs to distinguish between immediate and latent IGP risks^1^,^7^,^8^,^26^. Since every anti-predator behavior has costs, misjudging the risks associated with a given predator or cue would have negative consequences for prey. Thus, prey is expected to behave threat-sensitively in predator avoidance^3^,^4^ and this is exactly what the predator-experienced protonymphs from stressed mothers did in our experiments. They avoided overreaction and saved energy by being less restless in presence of predator traces alone. We argue that the observed behavioral changes represent optimized risk management, as has been shown for various animals including fish, salamanders and spider mites^26^,^27^,^28^, i.e. an attenuated response to alarm and/or predator traces following physical predator experience. We did not test whether the changed behavior occurs, and is adaptive, under all IGP circumstances and transitions between IGP risk levels, respectively, or not. For example, if the protonymphs would respond less to physically present predators, one could argue that the maternal effects compromised their sensitivity to predator cues, as shown for sticklebacks^29^. However, while the predators in the stickleback study posed an extremely high risk^29^, in our study the IG predators pose only a minor, if not negligible, risk to the age-advanced *P. persimilis* protonymphs. Thus, we conclude that *P. persimilis* mothers experiencing IGP risk adaptively influence their offspring to behave more optimally in IGP environments. Our study is a key example for maternal effects in IGP and learning scenarios.

## Additional Information

**How to cite this article**: Seiter, M. and Schausberger, P. Maternal intraguild predation risk affects offspring anti-predator behavior and learning in mites. *Sci. Rep.* **5**, 15046; doi: 10.1038/srep15046 (2015).
